# Ocular Findings in the 16p11.2 Microdeletion Syndrome: A Case Report and Literature Review

**DOI:** 10.1155/2020/2031701

**Published:** 2020-04-20

**Authors:** Cybil S. Stingl, Colleen Jackson-Cook, Natario L. Couser

**Affiliations:** ^1^Virginia Commonwealth University School of Medicine, Richmond, VA, USA; ^2^Department of Human and Molecular Genetics, Virginia Commonwealth University School of Medicine, Richmond, VA, USA; ^3^Department of Pathology, Virginia Commonwealth University School of Medicine, Richmond, VA, USA; ^4^Department of Ophthalmology, Virginia Commonwealth University School of Medicine, Richmond, VA, USA; ^5^Department of Pediatrics, Virginia Commonwealth University School of Medicine, Richmond, VA, USA

## Abstract

The recurrent 16p11.2 microdeletion is characterized by developmental delays and a wide spectrum of congenital anomalies. It has been well reported that individuals with this ∼593-kb interstitial deletion have an increased susceptibility toward the autism spectrum disorder (ASD). Abnormalities of the eye and ocular adnexa are also commonly associated findings seen in individuals with the 16p11.2 microdeletion syndrome, although these ophthalmic manifestations have not been well characterized. We conducted an extensive literature review to highlight the eye features in patients with the 16p11.2 microdeletion syndrome and describe a 5-year-old boy with the syndrome. The boy initially presented with intellectual disability, speech delay, and defiant behavior; diagnoses of attention deficit hyperactivity disorder (ADHD) and oppositional defiant disorder (ODD) were established. He had a Chiari malformation type 1. His ophthalmic features included strabismus, hyperopia, and ptosis, and a posterior embryotoxon was present bilaterally. From a systematic review of prior reported cases, the most common eye and ocular adnexa findings observed were downslanting palpebral fissures, deep-set eyes, ptosis, and hypertelorism.

## 1. Introduction

The 16p11.2 microdeletion syndrome (OMIM # 611913) is a rare congenital condition with an estimated frequency of 3 in 10,000 in the general Icelandic public [1]. The microdeletion is also present in 1 in 100 persons with autism and 1 in 1,000 persons with a language or psychiatric disorder, most notably speech delay and schizophrenia [1–3]. The syndrome most classically involves a heterozygous microdeletion of about 593-kb from band 16p11.2, which is localized to the short arm (*p*) of chromosome 16 [4]. A majority of cases reported are de novo, but the deletion is inherited in an autosomal dominant fashion from a parent 20% of the time [4]. An equal sex ratio has been reported [4]. The 16p11.2 microdeletion syndrome is characterized by the presence of developmental delays related to expressive language skills, autism spectrum disorder (ASD), diminished cognitive function, macrocephaly, hypotonia, Chiari malformation, learning disabilities, and high risk of encopresis and obesity [3, 4]. Seizures are reported in approximately 20% of individuals affected [4]. Reported cases of 16p11.2 microdeletion have included variable ocular findings [5–25]. We conducted an extensive literature review to summarize the eye features in patients with the microdeletion syndrome reported, to date, and describe a new case of a 5-year-old boy with the 16p11.2 microdeletion syndrome.

## 2. Case Report

Our patient, now a 5-year-old boy, was born at 36 5/7 weeks via vaginal delivery as the second child to a mother with a pregnancy complicated by subchorionic hematoma and bleeding at 17 weeks; birth weight was 6 lbs 13 oz. Tobacco use (1/2 pack per day) was present for the first 3 months of pregnancy. Paternal family history was notable for the presence of multiple personality disorder, depression, and schizophrenia.

Additional medical problems of the patient included significant speech and language delay, hypotonia with gait abnormality, encopresis, incontinence, hyperphagia with elevated BMI (85th percentile), attention deficit hyperactivity disorder (ADHD), oppositional defiant disorder (ODD), and Chiari malformation type 1.

**Figure 1:**
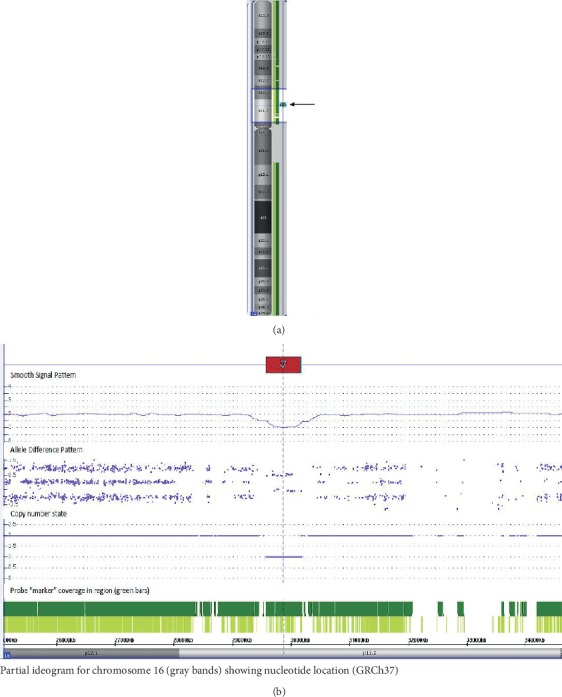
Deletion of 16p11.2 noted using microarray technology. (a) An ideogram of chromosome 16 shows the location of the deletion that is present in this patient in the context of the entire chromosome 16 (arrows). (b) The area highlighted (lighter colored region) in portion (a) of this figure is shown in an expanded view in this image. The deletion in this patient (highlighted by the red arrow; top row) is shown via smooth signal and allele difference patterns, as well as copy number state values. This deletion is localized to band 16p11.2 (nucleotides 29567295_30177999) [GRCh37].

Chromosomal microarray revealed a pathogenic microdeletion at 16p11.2(29567295_30177999)x1 (Figure 1). Parental testing revealed the mother was negative for the 16p11.2 microdeletion. The father was deceased and thus was not available for genetic testing.

**Figure 2:**
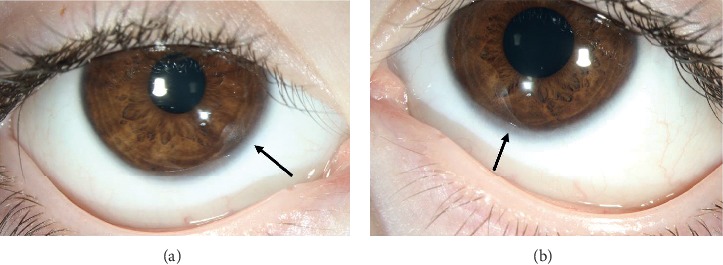
External photograph of the (a) right and (b) left eye showing the posterior embryotoxon (arrows).

At his most recent exam at 5 years and 3 months of age, ocular abnormalities included strabismus (intermittent exodeviation), bilateral mild hyperopia, ptosis of the left eyelid, and posterior embryotoxon present bilaterally (Figure 2).

## 3. Methods

We performed a systematic review of the literature to summarize the reported ocular and ophthalmic features in individuals with confirmed 16p11.2 microdeletions. A PubMed search of “16p11.2 Microdeletion Syndrome” led to a total of 44 articles; these articles, along with their references, were reviewed in search of ocular findings. Additionally, all 27 references used for the OMIM 16p11.2 microdeletion syndrome entry (MIM # 611913) were reviewed, along with their references. There were reports on an estimated 140 patients with the 16p11.2 microdeletion syndrome; 42 patient reports included descriptions of associated eye features (Table 1). The LeBlanc and Nelson study with 19 genetically confirmed 16p11.2 microdeletion patients was excluded from this analysis due to a lack of eye and ocular adnexa descriptions for their probands [25]. After including our patient, we calculated the frequency and prevalence of each ocular finding from the total number of cases with a confirmed deletion and reported eye findings (Table 2). No articles were excluded based on year published, and one article was excluded based on language.

## 4. Discussion

A systematic literature review revealed a total of 42 patients with confirmed 16p11.2 microdeletion and reported ophthalmic findings (Table 1) [5–25]. After including our patient, we calculated the frequency and prevalence of each ocular finding out the 43 patients with a confirmed deletion and reported eye findings (Table 2).

The 16p11.2 microdeletion syndrome was first reported in association with multiple congenital anomalies in 2002 in a boy who died at 5 months of age due to cardiac failure. The ocular findings of blepharophimosis, coloboma, and unilateral chorioretinitis were all reported in this initial case [10]. In 2007, data regarding facial dysmorphism in 16p11.2 microdeletion syndrome were published by Ballif et al. The dysmorphic features of 5 patients with confirmed microdeletions of 16p11.2 included the ocular findings of palpebral fissures, epicanthal folds, deep-set eyes, absent tear ducts, strabismus, hypo- and hypertelorism, and hyperopia [19]. In 2008, additional information regarding facial dysmorphism in a boy with 16p11.2 microdeletion was published by Kumar et al. This report consisted of downslanting palpebral fissures, prominent ears, and broad nasal root [12]. In 2009, Shimojima et al. reported the case of a 3-year-old boy with a confirmed 16p11.2 microdeletion. This boy was described with mildly dysmorphic features, bilateral ptosis, developmental delay, speech delay, hemivertebrae, early onset obesity, and dilation of the lateral ventricles on brain MRI [16]. Since then, there have been reports on over 130 patients with documented 16p11.2 microdeletions; the majority of these reports lack ophthalmic descriptions. It is possible these patients without reported eye manifestations did not have abnormal eye or ocular adnexa features, although we suspect the omission of reported eye features was more frequently related to the particular concentration of topics within each individual report. For example, an article titled “The copy number variation landscape of congenital anomalies of the kidney and urinary tract” by Miguel Verbitsky et al. describes extrarenal malformations in a variety of patients with copy number variants (CNV), including 9 patients with confirmed 16p11.2 microdeletions; ophthalmic manifestations were not featured [26].

Of the reports that included ophthalmic descriptions, the most common eye feature reported was abnormal palpebral fissures (*n* = 18, freq = 41.9%). Downslanting was the most common description of palpebral fissures (*n* = 12, freq = 66.7%), along with short (*n* = 6, freq = 33.3%) and narrow (*n* = 5, freq = 27.8%). Less commonly, there was one report of upslanting (*n* = 1, freq = 5.6%) and one report of long (*n* = 1, freq = 5.6%) palpebral fissures. The next most common ocular findings following abnormal palpebral fissures was deep-set eyes (*n* = 9, freq = 20.9%). This was followed by both ptosis and hypertelorism (*n* = 8, freq = 18.6%) (Table 2). Epicanthal folds and strabismus (*n* = 4, freq = 9.3%) were also common ocular findings. Infrequent reportings were notable for retinitis pigmentosa, coloboma, blepharophimosis, hypotelorism, hyperopia, and full lateral part of the upper eyelids (*n* = 2, freq = 4.7%). Isolated ophthalmic features included iris heterochromia, thick supraorbital ridge, microphthalmia, chorioretinitis, prominent eyes, horizontal nystagmus, retinal dystrophy, absent tear ducts, eyelid eversion, long eyelashes, sagging lateral upper eyelids, prominent infraorbital creases, epicanthus inversus, and telecanthus (*n* = 1, freq = 2.3%).

In summary, this report and literature review of the 16p11.2 microdeletion syndrome contributes to our understanding of the relationship of the 16p11.2 microdeletion and ocular abnormalities and provides incentive to further investigate the phenotype. A limitation of our study was the inability to directly compare the frequency of these ocular findings to their incidence in the general public. Further studies with a larger sample size could be beneficial to distinguish whether many of these features are indeed a true correlation with this microdeletion syndrome. We also recommend further investigation on the role of 16p11.2 microdeletion in eye development to help elucidate the mechanisms underlying ophthalmologic and adnexa abnormalities seen in the syndrome.

## Figures and Tables

**Table 1:** Ocular findings by patient.

Patient	Year	Reference	Ophthalmic findings
1	2019	Stingl et al. (this report)	Intermittent exodeviation, mild hyperopia bilaterally, ptosis of the left eyelid, and a posterior embryotoxon bilaterally
2	2018	Dell'Edera et al. [8]	Hypertelorism (interpupillary distance of 2.9 cm), mildly downslanting palpebral fissures
3	2018	Gatti et al. [22]	Downslanting palpebral fissures, slight eversion of the lateral third of the lower eyelid, long eyelashes
4	2015	Moreno-Igoa et al. [13]	Short and upslanting palpebral fissures, hypertelorism, epicanthal folds, ptosis of the eyelids, iris heterochromia
5	2014	D'Angelo et al. [7]	Deep-set eyes
6	2014	Pebrel-Richard et al. [14]	Acute visual impairment appeared with retinitis pigmentosa and progressive visual loss
7	2014	Gerundino et al. [21]	Downslanting palpebral fissures with deep-set eyes
8	2012	Kino et al. [11]	Hypertelorism
9	2012	Tabet et al. [18]	Deep-set eyes, thick supraorbital ridge
10	2011	Barge-schaapveld et al. [6]	Patient 1: sagging lateral upper eyelids
11			Patient 4: Full lateral part of the upper eyelids
12			Patient 3: right-sided convergent strabismus, hypotelorism, narrow palpebral fissures, full lateral part of the upper eyelids, relatively thin eyebrows with lateral notches
13	2011	Schaaf et al. [15]	Prominent eyes
14			Mild horizontal nystagmus on extreme lateral gaze
15	2010	Bardakjian et al. [5]	Left microphthalmia, persistent hyperplastic primary vitreous and posterior coloboma, right posterior pole coloboma
16	2010	Shinawi et al. [9]	Mild hypertelorism
17			Downslanting palpebral fissures
18			Deep-set eyes
19			Hypertelorism
20			“Almond shaped” eyes
21			Hypertelorism
22	2010	Fernandez et al. [17]	Proband 2, 13yo boy: long, narrow palpebral fissures
23			proband3c (mother): deep-set eyes
24			Proband 3, 5 yo male: hypertelorism (inner canthal distance 3.4 cm, > +2SD)
25	2010	Sampson et al. [20]	Retinal dystrophy, retinitis pigmentosa
26	2009	Shimojima et al. [16]	Bilateral ptosis
27	2009	Hemple et al. [23]	Deep-set eyes
28	2009	Bijlsma et al. [24]	Short palpebral fissures
29			Short and down-slanted palpebral fissures
30			Hypertelorism, bilateral epicanthic folds, short palpebral fissures, mild ptosis
31			Prominent infraorbital skin creases
32			Slightly deep-set eyes
33			Mild ptosis
34			Blepharophimosis, ptosis, epicanthus inversus, telecanthus
35			Downslanting and narrow palpebral fissures
36			Downslanting and narrow palpebral fissures
37	2008	Kumar et al. [12]	Downslanting palpebral fissures
38	2007	Ballif et al. [19]	Downslanting palpebral fissures; bilateral epicanthal folds; deep-set eyes; absent tear ducts; strabismus
39			Short and downslanting palpebral fissures; relative hypotelorism (3rd–10th percentile)
40			Downslanting palpebral fissures; left epicanthal fold; hypotelorism
41			Downslanting palpebral fissures; mild epicanthal folds; deep-set eyes
42			Narrow and slightly short palpebral fissures; relative hypertelorism (0 to +1SD); ptosis (right eye); strabismus (left eye); hyperopia
43	2002	Hernando et al. [10]	Blepharophimosis, coloboma and unilateral chorioretinitis (right eye)

**Table 2:** Frequency of ocular findings.

Ocular findings	Number (*n*)	Frequency (%)
Abnormal palpebral fissures	18	41.9
^*∗*^Downslanting	12	66.7
^*∗*^Upslanting	1	5.6
^*∗*^Narrow	5	27.8
^*∗*^Short	6	33.3
^*∗*^Long	1	5.6
Deep-set eyes	9	20.9
Ptosis	8	18.6
Hypertelorism	8	18.6
Epicanthal folds	4	9.3
Strabismus	4	9.3
Retinitis pigmentosa	2	4.7
Coloboma	2	4.7
Blepharophimosis	2	4.7
Hypotelorism	2	4.7
Hyperopia	2	4.7
Full lateral part of the upper eyelids	2	4.7
Iris heterochromia	1	2.3
Thick supraorbital ridge	1	2.3
Microphthalmia	1	2.3
Chorioretinitis	1	2.3
Prominent eyes	1	2.3
Horizontal nystagmus	1	2.3
Retinal dystrophy	1	2.3
Absent tear ducts	1	2.3
Eyelid eversion	1	2.3
Long eyelashes	1	2.3
Sagging lateral upper eyelids	1	2.3
Prominent infraorbital skin creases	1	2.3
Epicanthus inversus	1	2.3
Telecanthus	1	2.3
Posterior embryotoxon	1	2.3

^*∗*^Frequency within the subset of patients presenting with abnormal palpebral fissures.
